# Mycophenolate mofetil: an update on its mechanism of action and effect on lymphoid tissue

**DOI:** 10.3389/fimmu.2024.1463429

**Published:** 2025-01-07

**Authors:** Anna Krawczyk, Bernard Kravčenia, Tomasz Maślanka

**Affiliations:** Department of Pharmacology and Toxicology, Faculty of Veterinary Medicine, University of Warmia and Mazury in Olsztyn, Olsztyn, Poland

**Keywords:** Mycophenolate mofetil, mycophenolic acid, CD4^+^ T cells, CD8^+^ T cells, B cells, Treg cells, Foxp3, CD25

## Abstract

**Introduction:**

Mycophenolate mofetil (MMF) is an immunosuppressive drug administered in the management of both autoimmune diseases and organ transplantation. The main aims of the study were: (a) to obtain information regarding the safety of using MMF in respect of its effect on normal T and B cells in lymphoid tissues; (b) to investigate whether the generation of inducible Foxp3-expressing regulatory T cells (Treg) might constitute additional mechanisms underlying the immunosuppressive properties of MMF.

**Methods:**

The effect of MMF (*in vivo* studies) and its active metabolite, mycophenolic acid, (*in vitro* studies) on murine CD4^+^ and CD8^+^ T cells as well as B cells was determined, regarding: (a) absolute count, proliferation and apoptosis of these cells (*in vitro* studies); (b) absolute count of these cells in the head and neck lymph nodes, mesenteric lymph nodes and the spleen (*in vivo* studies).

**Results:**

The study found that the treatment of mice with MMF induced depletion of CD4^+^ and CD8^+^ T cells and B cells in lymph nodes and the spleen, and the magnitude of these effects should be considered as clinically relevant. The study demonstrated the following actions of the drug: (a) proapoptotic action on effector CD4^+^ and CD8^+^ T (Teff) cells, and B cells; (b) the down-regulation or prevention of activation-induced expression of CD25 on CD4^+^ and CD8^+^ Teff cells; (c) the generation of Foxp3^+^CD25^+^CD4^+^ and Foxp3^+^CD25^+^CD8^+^ T cells resulting in the shift of the Treg cell/activated Teff cell balance toward an increased proportion of Treg cells.

**Conclusion:**

The depletive effect of MMF on CD4^+^ and CD8^+^ T cells and B cells in lymphoid tissues should be taken into account when assessing the benefit-risk ratio of using MMF in patients with microbial infections and undergoing vaccination. The study contributed new insight into this mechanism, indicating that MMF effectively prevents the proliferation of T and B cells, but may be less effective against already proliferating cells. Moreover, the findings of the study strongly suggest the existence of additional mechanisms that may be responsible for the clinical efficacy of the drug, including the induction of Foxp3-expressing CD4^+^ and CD8^+^ Treg cells.

## 1 Introduction

Mycophenolate mofetil (MMF), an inactive prodrug of mycophenolic acid (MPA), is an immunosuppressive drug that was initially introduced for the treatment of the graft-versus-host disease in organ transplantation (GVHR) (1, 2). However, during the last twenty years, the indications for MMF have been extended to the treatment of autoimmune rheumatological diseases, such as systemic lupus erythematosus (SLE), systemic sclerosis, dermatomyositis, and systemic vasculitis (3). The active metabolite of MMF, MPA, is a selective, non-competitive, and reversible inhibitor of inosine monophosphate dehydrogenase (IMPDH), a key enzyme in the *de novo* synthesis of guanine nucleotides, which is preferentially expressed in activated lymphocytes (4). In this way, MPA induces inhibition of T and B lymphocyte proliferation and maturation, thereby causing the suppression of cellular and humoral immune responses (1, 2).

Immunosuppressive agents are a class of drugs that inhibit the abnormal immune response of the body usually by dampening both the humoral and cellular components of the immune system. However, we should acknowledge the fact that immunosuppression induced by immunosuppressive therapy can be a double-edged sword, i.e. it can increase the patient’s susceptibility to and severity of bacterial, viral, fungal, and protozoal infections, including opportunistic infections. In fact, in the Summary of Product Characteristics (SmPC) of products containing MMF (e.g. 5, 6), such infections are listed as possible adverse effects of the drug and their occurrence has been confirmed in multiple retrospective studies. These studies revealed that the incidence of MMF-induced infections ranged from a dozen to several dozen percent (7–10). Although MMF was introduced into clinical practice almost thirty years ago, both the SmPC of products containing MMF and available literature fail to elucidate what immune mechanisms are responsible for increased susceptibility of patients to infections. Considering the fact that the pathological (i.e. involved in the pathogenesis of autoimmune diseases and GVHR) T and B cells are the target cells for MMF action, we have hypothesized that the administration of the drug can also induce depletion of normal T and B cells in the lymphoid tissue.

Although the effect of MMF on immune system has been investigated in various aspects (mainly in terms of mechanisms underlying its therapeutic actions) (e.g. 11–17), there is a significant gap in our knowledge concerning research-based evaluation of the drug on T and B cells in the context of undesirable effects. In this context, it should be emphasized that, to the best of the author’s knowledge, there is no published data describing the effect of treatment with MMF on lymphocytes in lymph nodes or other lymphoid tissues. Certainly, we lack broad-scale research focusing on the following question: does the treatment with MMF produce a negative effect on normal T and B cells in the lymphoid tissue, and if it does, what is the magnitude of the problem and what mechanisms are behind it? Therefore, to assess the risk of a negative impact of MMF on these immunocompetent cells, we decided to conduct such studies. To achieve this aim, the effect of MMF (*in vivo* studies) or MPA (*in vitro* studies) on CD4^+^ and CD8^+^ T cells as well as B cells was determined, regarding: (a) absolute count, proliferation and apoptosis of these cells (*in vitro* studies); (b) absolute count of these cells in the head and neck lymph nodes (HNLNs), mesenteric lymph nodes (MLNs) and the spleen (*in vivo* studies). To sum up, the first scientific and practical aim of this study has been to obtain information regarding the safety of using MMF in respect of its effect on normal T and B cells in lymphoid tissues, which in turn can improve the benefit-risk assessment of using this immunosuppressive agent in patients, especially those with bacterial and viral infections.

Unquestionably, a substantial mechanism of action of MMF is its antiproliferative effect exerted via IMPDH inhibition. However, immunomodulatory and immunosuppressive drugs often have very complex mechanisms of action and interfere with the immune response at different stages and levels (18). In the available literature, there are reports suggesting the existence of other additional mechanisms underlying MMF-mediated immunosuppression (11–17). The results of certain studies suggest that one such mechanism could be the generation of inducible Foxp3^+^CD25^+^CD4^+^ T regulatory (iTreg) cells (15, 16), although some other studies contradict it (14, 19, 20). In this study, we hypothesized that MMF may generate Foxp3-expressing CD4^+^ and CD8^+^ iTreg cells through induction of Foxp3 expression in Foxp3-negative CD4^+^ and CD8^+^ T cells. In order to verify this hypothesis, the effect of MPA and MMF on Foxp3 expression in CD4^+^ and CD8^+^ T cells was assessed under *in vitro* and *in vivo* experiments, respectively. Hence, another aim of this study was to broaden the knowledge by verifying whether the generation of iTreg cells might constitute additional mechanisms underlying the immunosuppressive properties of MMF.

## 2 Materials and methods

### 2.1 Animals

The animals were housed and treated in accordance with the rules of the Local Ethics Commission for Animal Experiments in Olsztyn (affiliated to the National Ethics Commission for Animal Experimentation, Polish Ministry of Science and Higher Education). All of the procedures were approved by the Local Ethics Commission (Ethic permission No. 59/2020). The experiments were carried out on 12-week-old Balb/c mice. The mice were bred and maintained under standard lab conditions [12/12 h light/dark cycle, controlled temperature (21 +/- 2°C) and humidity (55+/- 5%), and ad libitum access to food and water] in the Animal Facility of the Faculty of Veterinary Medicine, University of Warmia and Mazury in Olsztyn.

### 2.2 Treatment protocol

Mice were randomly divided into two groups, i.e. the control and MMF groups (n = 5 per group/experiment overall; n = 10 per group). MMF (Sigma-Aldrich, Schelldorf, Germany) was solubilized in dimethyl sulfoxide (DMSO; Sigma-Aldrich) and administered via intraperitoneal injection at a dose of 200 mg/kg once daily for 14 consecutive days. The dose was chosen based on an equivalent mouse dosage value converted from a human dosage of 2000 mg/day, using equations presented by Ng (21), i.e. following the procedure which was explained in detail by Richez et al. (22). Mice from the control group were treated with an equivalent volume of DMSO, which was used as a vehicle to dissolve MMF.

### 2.3 Isolation of lymphocytes – *in vivo* study

At 24 h after the last dose, the mice were euthanized by asphyxiation with CO_2_ and the head and neck lymph nodes (HNLNs; submandibular gland, parotid gland, deep cervical lymph nodes), the mesenteric lymph nodes (MLNs) and spleen were individually collected and subjected to dounce homogenization (23). The resulting cell suspensions were filtered through Nitex fabric (Fairview Fabrics, Hercules, CA, USA), washed with FACS (fluorescence-activated cell-sorting) buffer (FB; Dulbecco’s PBS devoid of Ca^2+^ and Mg^2+^ with 2% v/v heat ***-*** inactivated FBS, both from Sigma ***-*** Aldrich), and centrifuged (300 x g for 5 min. at 5˚C; the same parameters were used for all cell-washing procedures). Subsequently, the cells were re-suspended in FB, transferred into FACS tubes, counted and washed again with FB.

### 2.4 Isolation of lymphocytes – *in vitro* studies

For *in vitro* studies, cells were isolated, as described above, from the HNLN, MLNs, axillary lymph nodes and the spleen of untreated mice. The resulting cell suspensions were re-suspended in complete medium [CM; RPMI 1640, 10% FBS, 10 mM HEPES buffer, 10 mM nonessential amino acids, 10 mM sodium pyruvate and 10 U/ml penicillin/streptomycin (all from Sigma-Aldrich)], counted and seeded.

### 2.5 Culture conditions and cell harvesting

In each experiment, cells were exposed to MPA, i.e. the active metabolite of MMF, in concentrations reflecting its plasma level achievable *in vivo* at a typical dose (Product information for Myfortic) and in a ten-fold lower concentration (i.e. in concentrations of 10^-4^ and 10^-5^ M, respectively). MPA was dissolved in methanol; therefore, the same amount of methanol was added to control wells. The cells were adjusted to a final concentration of 5 x 10^6^ cells/mL in CM, seeded in 24-well plates in 1 mL aliquots and cultured for the following time, depending on the assay type: (a) to analyze the effect of MPA on apoptosis of T and B cells: 24 and 48 h; (b) to analyze the effect of MPA on the absolute count of T and B cells: 48 and 96 h both types of experiments were performed under unstimulated conditions; (c) to analyze the effect of MPA on the relative and absolute counts of IFN-γ-producing T cells: 18 h without stimulation, followed by 6 h stimulation with phorbol-12-myristate-13-acetate (PMA; 50 ng/ml) and ionomycin (1 µg/ml; both from Sigma-Aldrich) in the presence of brefeldin A (Protein transport inhibitor, 1 µl/ml; BD Biosciences) during last 4 h; (d) to analyze the effect of MPA on the relative and absolute counts of CD4^+^ and CD8^+^ Treg and activated effector T (aTeff) cells: 72 h including concomitant stimulation with IL-2 (Recombinant mouse IL-2, 20 ng/mL) and anti-CD3/anti-CD28 monoclonal antibodies (mAbs) (Purified NA/LE hamster anti-mouse CD3e, 1 μg/mL, clone 145-2C11; Purified NA/LE hamster anti-mouse CD28, 1 μg/mL, clone 37.51; all reagents from BD Biosciences, San Jose, USA) (24).

To analyze the proliferation rate of the studied cells, cells were adjusted to the final concentration of 2 x 10^6^ cells/mL in CM, seeded in 24-well plates in 1 mL aliquots and cultured for 72 h including concomitant stimulation with IL-2 and anti-CD3/anti-CD28 mAbs (as described above), and re-stimulation with PMA and ionomycin (as described above) for the last 3 h. The cells were exposed to MPA (10^-4^ M and 10^-5^ M) for the whole culture period (i.e. 72 h) or for the last 12 h. Proliferation was determined following the pulsing with 5-bromo-2’-deoxyuridine (BrdU; a final concentration of 100 µM in cell culture medium; APC BrdU Flow Kit, BD Biosciences) for the last 12 h.

Each experiment included 5 wells of lymphocytes (obtained from individual mice) for each setting tested. To eliminate the influences of individual differences between the animals, the same cells were used as both control and treated cells. All experiments were repeated independently two or three times. The plates were incubated at 37 °C in an atmosphere of humidified incubator with 5% CO_2_ and 95% air. Cells were removed from the wells by pipetting and rinsing with FB, and then transferred into FACS tubes for centrifugation. Subsequently, the cells were re-suspended in FB, counted and washed again with FB.

### 2.6 Flow cytometry

#### 2.6.1 Extracellular staining

Cell samples prepared as described above were pre-treated with anti-CD16/CD32 (clone: 2.4G2) FcR blocker for 15 minutes on ice and then stained for surface antigens with the following combinations of anti-mouse fluorochrome conjugated mAbs (1): FITC-anti-CD4 (clone H129.19; IgG2a, κ), APC-Cy7 anti-CD8a (clone 53-6.7; IgG2a, κ) and AF-700-anti-CD19 (clone 1D3; IgG2a, κ); (2) FITC-anti-CD4 (clone H129.19; IgG2a, κ) and APC-Cy7 anti-CD8a (clone 53-6.7; IgG2a, κ); (3) PerCP-Cy 5.5-anti-CD4 (clone RM4-5; IgG2a, κ), APC-Cy7-anti-CD8 (clone 53-6.7; IgG2a, κ) and BV605-anti-CD25 (clone PC61; IgG2a, κ all from BD Biosciences). After 30 min of incubation (on ice and in the dark), the cells were washed in 2 mL of FB.

#### 2.6.2 Staining for apoptosis evaluation

Following extracellular staining with the first mAb combination, the cells were washed once in 1 mL of 1x Annexin V binding buffer (BD Biosciences). The supernatants were removed by centrifugation and the cells were suspended in 1x Annexin V binding buffer and stained with of PE-conjugated Annexin V and 7-AAD (both from BD Biosciences). The cells were mixed gently and incubated for 15 min [at room temperature (RT) in the dark], and then diluted with 1x Annexin V binding buffer and analyzed by flow cytometry within 1 h.

#### 2.6.3 Intracellular staining for determination of IFN-γ-producing cells

Following extracellular staining with the second mAb combination, the cells were fixed and permeabilized using Cytofix/Cytoperm solution and Perm/Wash buffer (both from BD Biosciences) according to the manufacturer’s protocol. Subsequently, the cells were stained with APC-conjugated anti-IFN-γ mAb (clone XMG1.2; IgG1,κ; BD Biosciences). After 45 min of incubation (at RT in the dark), the cells were washed twice with 2 mL of FB and analyzed by flow cytometry.

#### 2.6.4 Staining for determination of Foxp3-expressing cells

Following extracellular staining with the third mAb combination, the cells were washed, fixed and permeabilized using a mouse Foxp3 buffer set (BD Biosciences) according to the manufacturer’s protocol. Subsequently, the cells were stained with AF-488-conjugated anti-Foxp3 mAb (clone MF23; IgG2b; BD Biosciences). After 45 min of incubation (at RT in the dark), the cells were washed twice with 2 mL of FB and analyzed by flow cytometry.

#### 2.6.5 Intracellular staining for determination of proliferating cells

Following extracellular staining with the first mAb combination, the cells were fixed, permeabilized and labeled for incorporated BrdU according to the manufacturer’s protocol (APC BrdU Flow Kit, BD Biosciences).

### 2.7 FACS acquisition and data analysis

Flow cytometry analysis was performed using a FACSCelesta cytometer (BD Biosciences). The data were acquired by FACSDiva version 9.0 software (BD Biosciences) and analyzed by FlowJo software (Tree Star Inc., Stanford, USA). Absolute cell count was obtained using the dual platform method. Briefly, the total cell count was calculated (using a cell counting chamber) for the whole HNLNs, MLNs and spleen harvested from individual mice (*in vivo* studies) and for individual wells of cell culture (*in vitro* studies). The absolute counts of evaluated cell subsets were determined by recalculating these data by the percentage of particular cell subsets (data from flow cytometry analysis), as illustrated in **Supplementary Figure S1**. Thus, the absolute count represented the number of cells from a particular subset per organ (*in vivo* studies) or per well (*in vitro* studies). Florescence minus one (FMO) controls (including isotype-matched mAbs) were used to confirm the gating strategy applied to identify Foxp3^+^CD25^+^ and Foxp3^-^CD25^+^ T cells. In turn, non-stimulated controls (including isotype-matched mAbs) were used to identify boundaries of gates for IFN-γ-producing and BrdU-incorporating cells.

### 2.8 Statistical analysis

Results were expressed as the mean (± S.D.) of two or three independent experiments. Statistical analysis was performed using one-way analysis of variance followed by the Bonferroni’s *post hoc* test (*in vitro* studies) or Student’s unpaired t-test (*in vivo* studies). Differences were deemed significant when the p values were < 0.05. SigmaPlot Software Version 12.0 (Systat Software Inc., San Jose, USA) was used for statistical analysis and the plotting of graphs.

## 3 Results

### 3.1 Both administration of MMF to mice and exposure of cell cultures to MPA induce the loss of CD4^+^ and CD8^+^ T cells, and CD19^+^ cells

**Figure 1:**
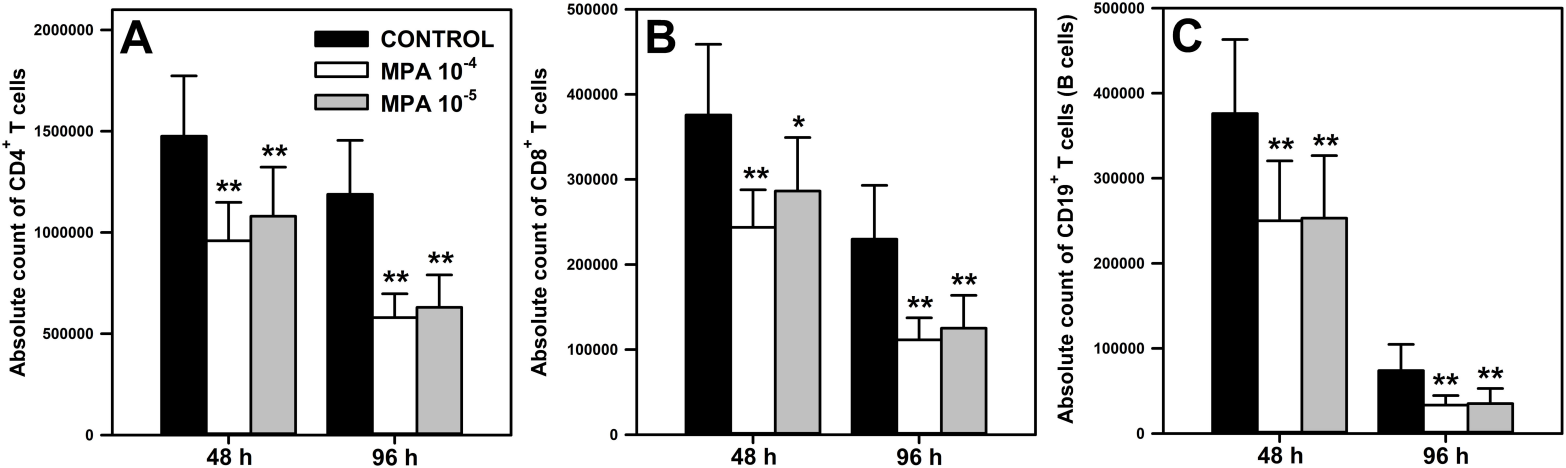
Effect of mycophenolic acid (MPA) on the absolute count of CD4^+^ and CD8^+^ T cells, and CD19^+^ cells under *in vitro* conditions. The parameters were determined in cell cultures incubated in the absence (CONTROL) and presence of MPA (10^-4^ M and 10^-5^ M) for 48 and 96 h The absolute count represents the number of CD4^+^ **(A)** and CD8^+^ **(B)** T cells, and CD19^+^ **(C)** cells per well. Results are presented as the mean (± S.D.) of three independent experiments with 5 wells per experiment (overall n = 15, *P = 0.002, ** P ≤ 0.001).

**Figure 2:**
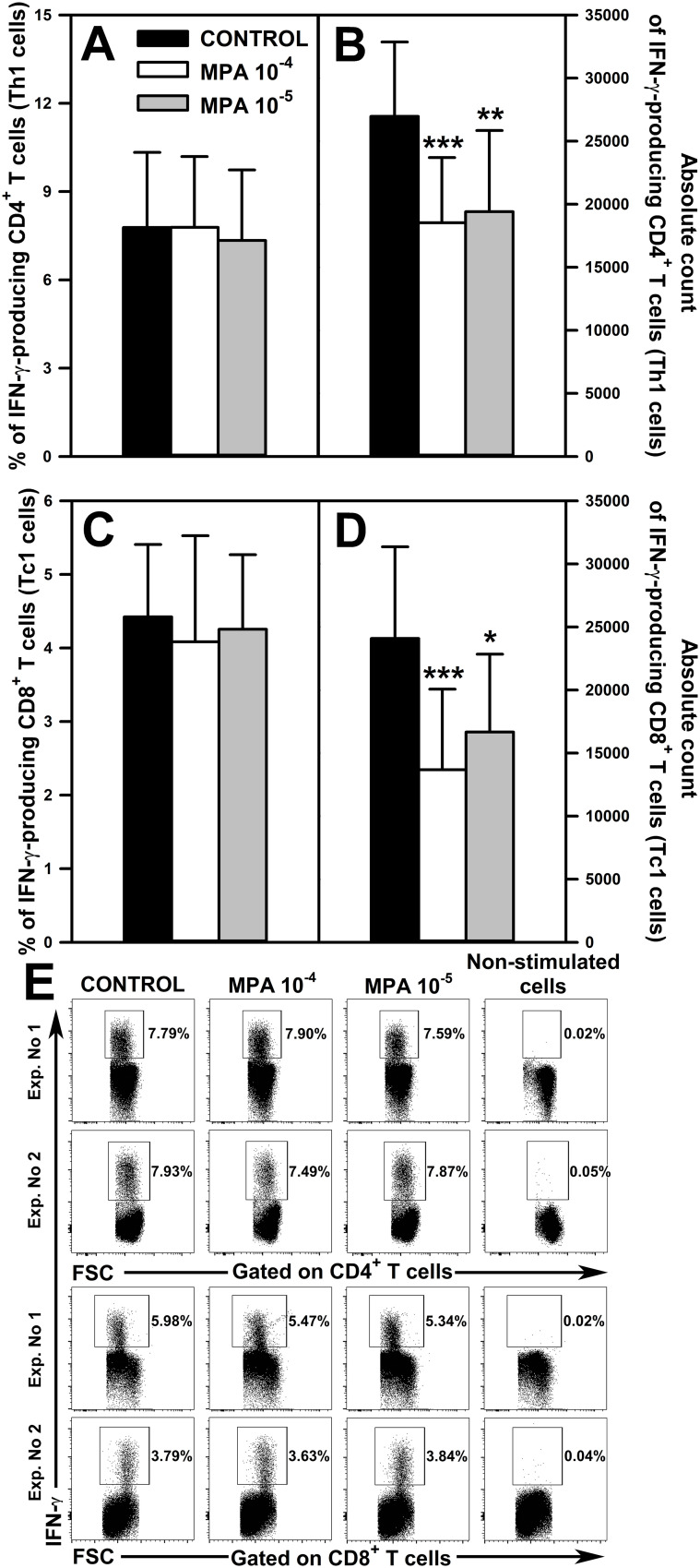
Effect of mycophenolic acid (MPA) on the relative and absolute counts of IFN-γ-producing CD4^+^ and CD8^+^ T cells. The parameters were determined in cell cultures incubated in the absence (CONTROL) and presence of MPA (10^-4^ M and 10^-5^ M) for 18 h followed by 6 h stimulation with phorbol-12-myristate-13-acetate and ionomycin in the presence of brefeldin A during last 4 h The relative count is expressed as a percentage of IFN-γ-producing cells within the CD4^+^ **(A)** and CD8^+^ **(C)** T cell subsets. The absolute count represents the number of IFN-γ^+^CD4^+^ **(B)** and IFN-γ^+^CD8^+^ **(D)** T cells per well. Cells with such phenotypes should be equated with Th1 and Tc2 cells, respectively. Results are presented as the mean (± S.D.) of three independent experiments with 5 wells per experiment (overall n = 15, *P < 0.05, **P < 0.01, ***P ≤ 0.001). Examples of dot plot cytograms showing the distribution of IFN-γ-producing cells within the CD4^+^ (**E**, upper panel) and CD8^+^ (**E**, lower panel) T cell subsets. Non-stimulated cells served as a negative control for the cell activation, and also were applied to confirm the gating strategy used to identify IFN-γ^+^ cells.

The effect of MPA on the absolute count of CD4^+^ (**Figure 1A**) and CD8^+^ (**Figure 1B**) T cells, and CD19^+^ cells (**Figure 1C**) was evaluated after 48 and 96 h of exposure of cells to the agent. In both time points, the exposure of cells to MPA in either of the two concentrations caused a significant reduction of the absolute counts of all studied subsets. The statistical analysis did not find any significant differences in terms of these parameters between both concentrations of MPA (**Figures 2A–C**). The mean depletion of CD4^+^ and CD8^+^ T cells, and CD19^+^ cells in samples obtained from cultures exposed to MPA 10^-4^ M for: (a) 48 h was 516147 ± 211498, 132073 ± 63225 and 125987 ± 83575 cells, respectively, which corresponds to 34.98%, 35.15% and 33.51% of the respective control values; (b) 96 h was 608640 ± 202618, 118433 ± 47459 and 40554 ± 27329 cells, respectively, which corresponds to 51.23%, 51.53 and 54.91% of the respective control values. In turn, the mean depletion of CD4^+^ and CD8^+^ T cells, and CD19^+^ cells in samples obtained from cells cultured in the presence of MPA 10^-5^ M for: (a) 48 h was 395573 ± 287955, 89373 ± 85855 and 122953 ± 65513 cells, respectively, which corresponds to 26.81%, 23.79% and 32.70% of the adequate control values; (b) 96 h was 557467 ± 215666, 104793 ± 45708 and 38698 ± 23426 cells, respectively, which corresponds to 46.92%, 45.59% and 52.40% of the respective control values. The mean depletion of CD4^+^ and CD8^+^ T cells, and CD19^+^ cells is an averaged (± SD) difference in the absolute count of these cells between the control and treated samples for a given time point. This parameter is expressed as a percentage of adequate control cells.

Among effector T cells, T helper type 1 (Th1) and T cytotoxic type 1 (Tc1) cells are crucial for cell-mediated immune responses (mainly toward intracellular pathogens and tumors), and are characterized by the production of the signature cytokine IFN-γ. Considering the above, it was deemed as justifiable and essential to evaluate the safety of MMF in respect of its effect on the ability of Th1 and Tc1 cells to produce IFN-γ and their absolute count. No effect of MPA on the percentage of IFN-γ-producing cells within both CD4^+^ (**Figure 2A** and upper panel of **Figure 2E**) and CD8^+^ (**Figure 2C** and lower panel of **Figure 2E**) T cell subsets was observed. However, the research demonstrated that the exposure of cells to MPA in both concentrations caused a significant reduction in the absolute count of IFN-γ^+^CD4^+^ (**Figure 2B**) and IFN-γ^+^CD8^+^ (**Figure 2D**) T cells; cells with such phenotypes should be equated with Th1 and Tc1 cells, respectively. The mean depletion of IFN-γ^+^CD4^+^ and IFN-γ^+^CD8^+^ T cells in samples obtained from cultures exposed to MPA 10^-4^ M was 8435 ± 4708 and 10406 ± 5023 cells, respectively, which corresponds to 31.28% and 43.22% of the respective control values. In turn, the mean depletion of IFN-γ^+^CD4^+^ and IFN-γ^+^CD8^+^ T cells in samples obtained from cells cultured in the presence of MPA 10^-5^ M for was 7553 ± 5262 and 7416 ± 5700 respectively, which corresponds to 28.01% and 30.80% of the respective control values.

**Figure 3:**
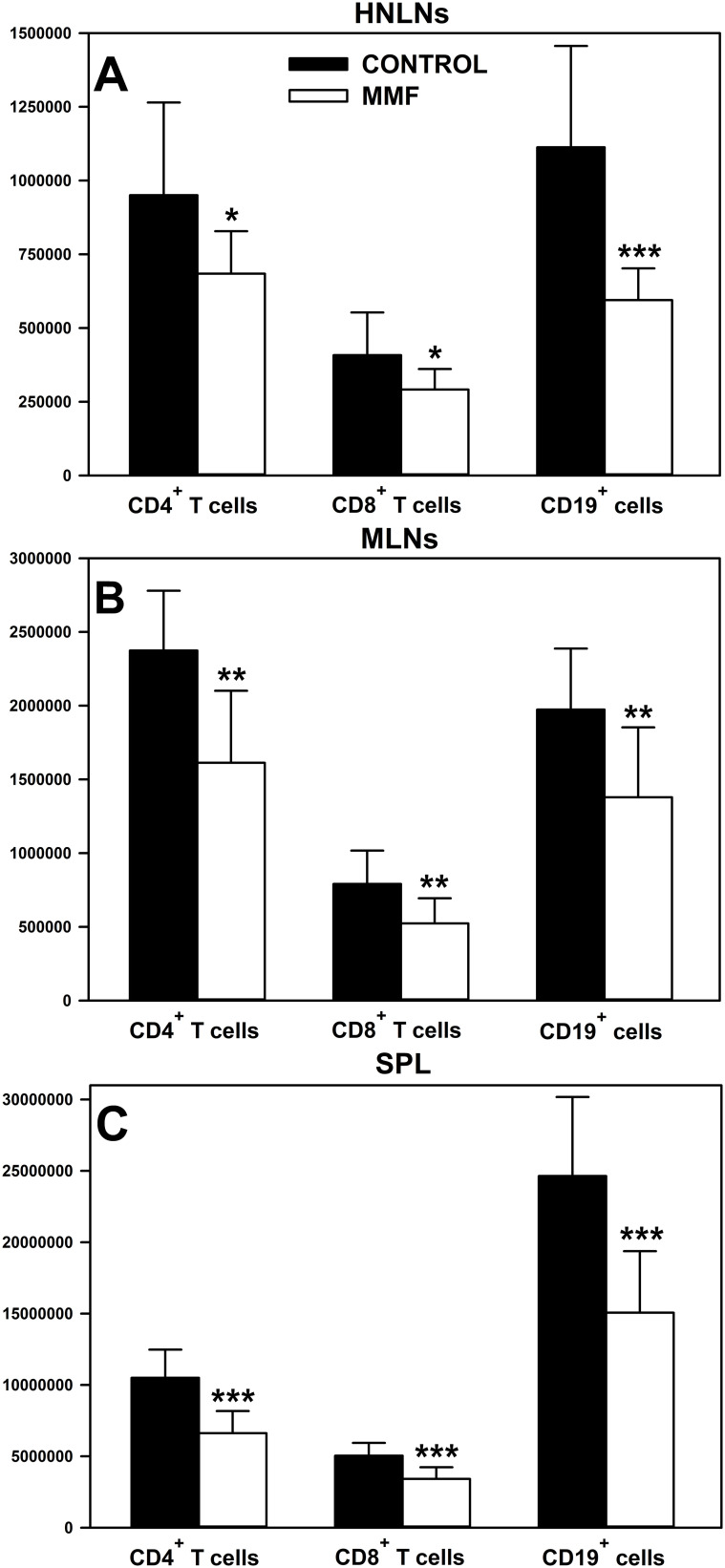
Effect of mycophenolate mofetil (MMF) on the absolute count of CD4^+^ and CD8^+^ T cells, and CD19^+^ cells in the head and neck lymph nodes (HNLNs), mesenteric lymph nodes (MLNs) and spleen (SPL) in mice. The absolute count represents the number of CD4^+^ and CD8^+^ T cells, and CD19^+^ cells in the whole HNLNs **(A)**, MLNs **(B)** and SPL **(C)** collected from non-treated (CONTROL) and MMF-treated (MMF) mice. Results are presented as the mean (± S.D.) of two independent experiments with 5 mice per group (n = 10 per group, *P < 0.05, **P < 0.01, ***P ≤ 0.001).

The *in vivo* study demonstrated that the treatment with MMF reduced the absolute count of CD4^+^ and CD8^+^ T cells, and CD19^+^ cells in the HNLNs (**Figure 3A**), MLNs (**Figure 3B**) and spleen (**Figure 3C**). The mean depletion of CD4^+^ and CD8^+^ T cells, and CD19^+^ cells in the HNLNs was 265975 ± 364827, 116725 ± 156048 and 518300 ± 417309 cells, respectively, which corresponds to 27.99, 28.61 and 46.57% of the respective control values. In turn, the mean depletion of CD4^+^ and CD8^+^ T cells, and CD19^+^ cells in the MLNs was 762155 ± 574651, 268065 ± 229325 and 594450 ± 624881 cells, respectively, which corresponds to 32.10%, 33.88% and 30.13% of the adequate control values. As regards the spleen, the mean depletion of CD4^+^ and CD8^+^ T cells, and CD19^+^ cells in this organ was 3886800 ± 1451132, 1626120 ± 1287926 and 9573200 ± 5953353 cells, respectively, which corresponds to 37.00%, 32.26% and 38.85% of the respective control values.

### 3.2 The administration of MPA to extensively proliferating lymphocyte culture exerts only a weak antiproliferative effect on CD4^+^ and CD8^+^ T cells, and on CD19^+^ cells

**Figure 4:**
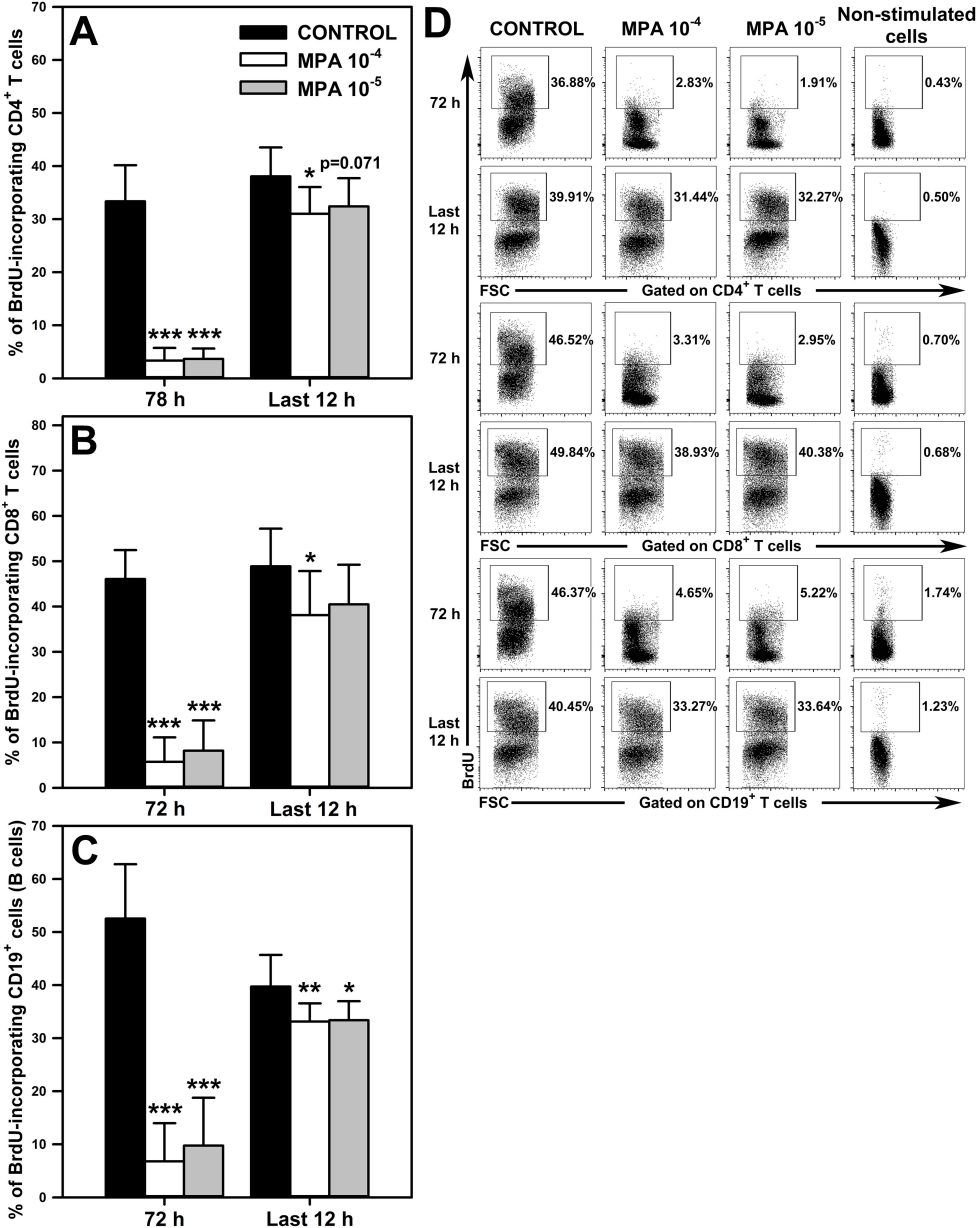
Effect of mycophenolic acid (MPA) on proliferation of CD4^+^ and CD8^+^ T cells, and CD19^+^ cells. The proliferation was assessed in cell cultures incubated in the absence (CONTROL) and presence of MPA (10^-4^ M and 10^-5^ M) for 72 h including concomitant stimulation with IL-2, anti-CD3/anti-CD28 antibodies, phorbol-12-myristate-13-acetate and ionomycin in the presence of bromo-2′-deoxyuridine (BrdU) during last 12 h Cultures were exposed to MPA for the whole culture period or for last 12 h The results are expressed as a percentage of BrdU-incorporating cells within the CD4^+^ **(A)**, CD8^+^ **(B)** and CD19^+^ **(C)** cell subsets, and they are presented as the mean (± S.D.) of two independent experiments with 5 wells per experiment (overall n = 10, *P < 0.05, **P < 0.01, ***P ≤ 0.001). Examples of dot plot cytograms showing the distribution of BrdU-incorporating cells within the CD4^+^ (**D**, upper panel), CD8^+^ (**D**, middle panel) and CD19^+^ (**D**, lower panel) cell subsets. Non-stimulated cells served as a negative control for the cell activation, and also were applied to confirm the gating strategy used to identify BrdU^+^ cells.

The effect of MPA on the proliferation of CD4^+^ and CD8^+^ T cells, and on CD19^+^ cells was determined in cell cultures stimulated for 72 h in the presence of BrdU during the last 12 h. The cells were exposed to MPA for the whole culture period or for the last 12 h (i.e. at the second time point MPA was administered to the cultures at the same time as BrdU). The exposure of cells to both concentrations of MPA for the whole culture period (i.e. 72 h) significantly reduced the percentage of BrdU-incorporating CD4^+^ (**Figure 4A** and upper panel of **Figure 4D**), CD8^+^ (**Figure 4B** and middle panel of **Figure 4D**) and CD19^+^ (**Figure 4C** and lower panel of **Figure 4D**) cells, and the statistical analysis did not find any significant differences between both concentrations of MPA in this respect. These effects can be regarded as spectacular because the mean percentage of BrdU-incorporating CD4^+^ and CD8^+^ T cells, and CD19^+^ cells in samples obtained from cultures exposed to MPA 10^-4^ M constituted only 10.02%, 12.43% and 12.94 of corresponding control values, respectively. Similarly, the mean percentage of BrdU-incorporating CD4^+^ and CD8^+^ T cells, and CD19^+^ cells in the cultures treated with MPA 10^-5^ M constituted only 10.98%, 17.80% and 18.56% of corresponding control values, respectively.

The study demonstrated that exposure of cells to MPA 10^-4^ M for the last 12 h of culture (i.e. the administration of the agent to extensively proliferating cell culture) statistically significantly reduced the percentage of BrdU-incorporating CD4^+^ (**Figure 4A** and upper panel of **Figure 4D**), CD8^+^ (**Figure 4B** and middle panel of **Figure 4D**) and CD19^+^ (**Figure 4C** and lower panel of **Figure 4D**) cells. However, the magnitude of this effect was very small compared to that found in the cultures exposed to MPA for the whole culture period. This assertion is based on the finding that the mean percentage of BrdU-incorporating CD4^+^ and CD8^+^ T cells, and CD19^+^ cells in samples obtained from cultures exposed to MPA 10^-4^ M constituted as much as 81.50%, 77.96% and 83.40% of corresponding control values, respectively. Treatment with MPA 10^-5^ M for the last 12 h of culture led to a significant decrease in the percentage of BrdU-incorporating CD19^+^ cells (**Figure 4C** and lower panel of **Figure 4D**), although this effect was also very weakly expressed as the mean percentage of these cells constituted as much as 84.02% of corresponding control values. Such treatment did not affect the percentage of BrdU-incorporating CD4^+^ (**Figure 4A** and lower upper panel of **Figure 4D**), and CD8^+^ T (**Figure 4B** and lower upper panel of **Figure 4D**) cells, although a certain trend (p = 0.071) toward decreasing this parameter with respect to CD4^+^ T cells was observed.

### 3.3 There is elevated apoptosis of CD4^+^ and CD8^+^ T cells, and CD19^+^ cells in the cultures exposed to MPA 10^-4^ M

**Figure 5:**
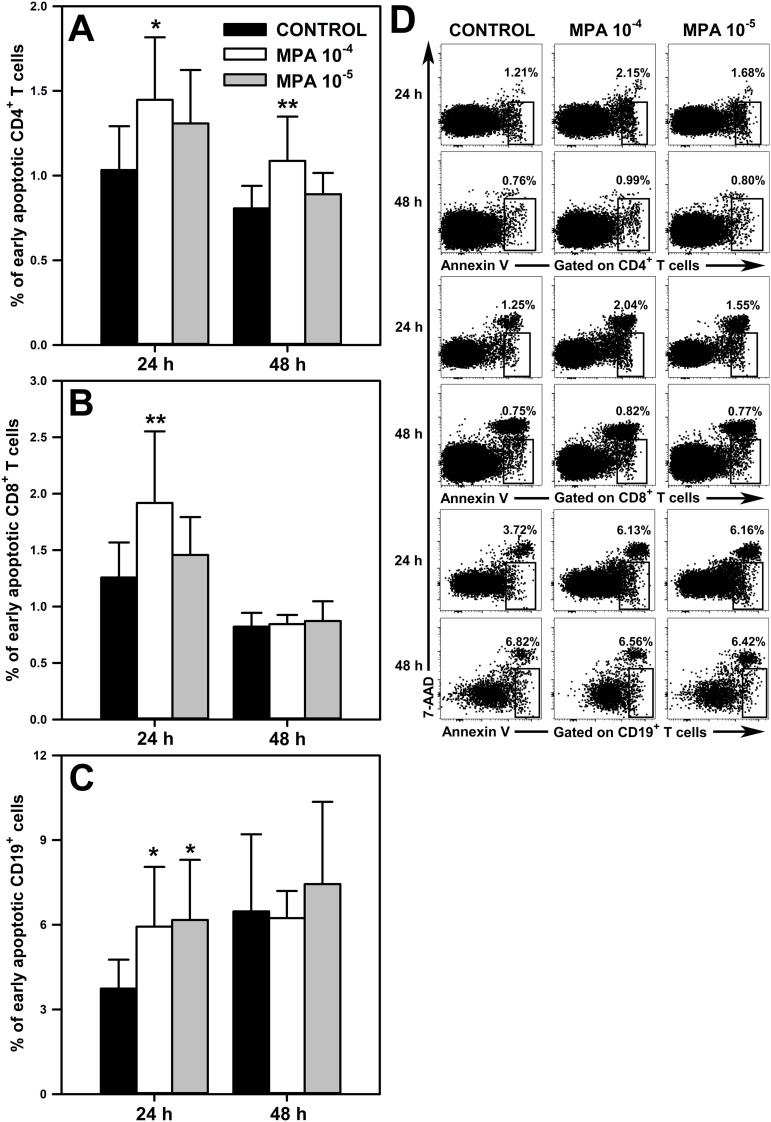
Effect of mycophenolic acid (MPA) on apoptosis of CD4^+^ and CD8^+^ T cells, and CD19^+^ cells. The apoptosis was assessed in cell cultures incubated in the absence (CONTROL) and presence of MPA (10^-4^ M and 10^-5^ M) for 24 and 48 h The results are expressed as a percentage of Annexin V^+^7-AAD^-^ (i.e. early apoptotic) cells within the CD4^+^ **(A)**, CD8^+^ **(B)** and CD19^+^ **(C)** cell subsets, and they are presented as the mean (± S.D.) of two independent experiments with 5 wells per experiment (overall n = 10, *P < 0.05, **P < 0.01). Examples of dot plot cytograms showing the distribution of Annexin V^+^7-AAD^-^ cells within the CD4^+^ (**D**, upper panel), CD8^+^ (**D**, middle panel) and CD19^+^ (**D**, lower panel) cell subsets.

A question arises about the mechanism behind the MMF/MPA-induced depletion of T and B cells revealed in our study. This effect demonstrated in unstimulated cell cultures must have been a consequence of increased cell death because such conditions exclude two other potential causes of cell depletion, namely the redistribution of cells or inhibition of their proliferation (lymphocytes practically do not proliferate under unstimulated conditions). Therefore, the effect of MPA on the apoptosis of CD4^+^ and CD8^+^ T cells, and CD19^+^ cells was assessed. Apoptosis assessment was performed after 24 and 48 h cell culture in the absence or presence of the agent. The study revealed a significant increase in the percentage of early apoptotic cells (i.e. Annexin V^+^7-AAD^-^ cells) within the CD4^+^ (**Figure 5A** and upper panel of **Figure 5D**), CD8^+^ T (**Figure 5B** and middle panel of **Figure 5D**) and CD19^+^ cell subsets (**Figure 5C** and lower panel of **Figure 5D**) exposed to MPA 10^-4^ M for 24 h. Moreover, it was found that the 48 h exposure to MPA 10^-4^ M increased the percentage of early apoptotic cells within CD4^+^ T cell pool (**Figure 5A** and upper panel of **Figure 5D**), but did not affect the percentage of these cells within the CD8^+^ T (**Figure 5B** and middle panel of **Figure 5D**) and CD19^+^ cell (**Figure 5C** and lower panel of **Figure 5E**) subsets. No significant effect of MPA at the lower concentration on the percentage of early apoptotic cells among CD4^+^ (**Figure 5A** and upper panel of **Figure 5D**) and CD8^+^ T cells (**Figure 5A** and middle panel of **Figure 5D**) was noticed. However, the study found an increased percentage of early apoptotic cells within CD19^+^ cell subsets in the cultures treated with MPA 10^-5^ M for 24 h (**Figure 5C** and lower panel of **Figure 5E**).

### 3.4 MPA increases the percentage of Foxp3^+^CD25^+^CD4^+^ and Foxp3^+^CD25^+^CD8^+^ T cells under activation conditions

**Figure 6:**
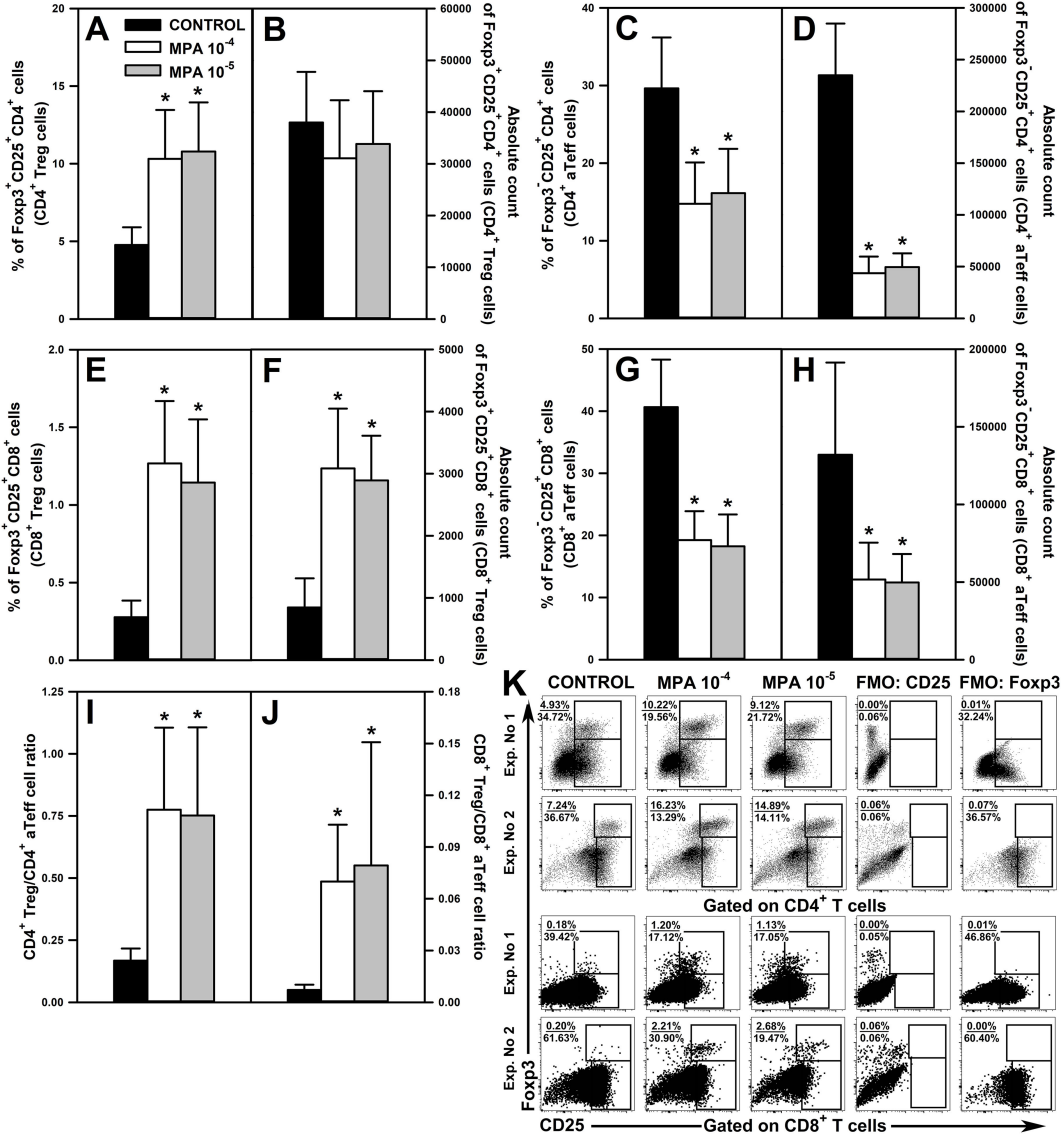
Effect of mycophenolic acid (MPA) on the relative and absolute counts of Foxp3^+^CD25^+^CD4^+^, Foxp3^+^CD25^+^CD8^+^ T regulatory (CD4^+^ and CD8^+^ Treg, respectively) cells and Foxp3^-^CD25^+^CD4^+^ and Foxp3^-^CD25^+^CD8^+^ activated effector T (CD4^+^ and CD8^+^ aTeff, respectively) cells under *in vitro* activation conditions. The parameters were determined in cell cultures incubated in the absence (CONTROL) and presence of MPA (10^-4^ M and 10^-5^ M) for 72 h including concomitant stimulation with IL-2 and anti-CD3/anti-CD28 antibodies. The relative count is expressed as a percentage of Foxp3^+^CD25^+^ and Foxp3^-^CD25^+^ cells within the CD4^+^ (**A, C**, respectively) and CD8^+^ (**E, G**, respectively) T cell subsets. The absolute count represents the number of Foxp3^+^CD25^+^CD4^+^ **(B)**, Foxp3^-^CD25^+^CD4^+^ **(D)**, Foxp3^+^CD25^+^CD8^+^ **(F)** and Foxp3^-^CD25^+^CD8^+^ **(H)** cells per well. Treg/aTeff ratio **(I, J)** was calculated by dividing the percentage of Treg cells by the percentage of aTeff cells. Results are presented as the mean (± S.D.) of three independent experiments with 5 wells per experiment (overall n = 15, *P < 0.001). Examples of dot plot cytograms showing the distribution of Foxp3^+^CD25^+^ and Foxp3^-^CD25^+^ cells within the CD4^+^ (**K**, upper panel) and CD8^+^ (**K**, lower panel) T cell subsets. Fluorescence minus one (FMO) controls were applied to confirm the gating strategy used to identify the Foxp3^+^CD25^+^ and Foxp3^-^CD25^+^ cell subsets.

The exposure of cells to MPA in both concentrations significantly increased the percentage of Foxp3^+^CD25^+^CD4^+^ (**Figure 6A** and upper panel of **Figure 6K**) and Foxp3^+^CD25^+^CD8^+^ Treg cells (**Figure 6E** and lower panel of **Figure 6K**) and the absolute count of the latter, but not the former, cell subset (**Figures 6B, F**). MPA-induced increases in the percentage of Foxp3^+^CD25^+^CD4^+^ and Foxp3^+^CD25^+^CD8^+^ Treg cells represented relatively large-scale effects as the mean values of these parameters were over 2- and 4-times greater, respectively, than the corresponding control values. Moreover, the absolute count of Foxp3^+^CD25^+^CD8^+^ Treg cells in the cultures treated with MPA was increased by about 3.5-times compared to control values. These results indicate that MPA under activation conditions increased the expression of Foxp3 in CD4^+^ and CD8^+^ T cells.

The treatment with both concentrations of MPA considerably reduced the percentage and absolute count of Foxp3^-^CD25^+^CD4^+^ (**Figures 6C, D** and upper panel of **Figure 6K**) and Foxp3^-^CD25^+^CD8^+^ aTeff cells (**Figures 6G, H** and lower panel of **Figure 6K**). The magnitude of these effects was also relatively large as the mean percentage of Foxp3^-^CD25^+^CD4^+^ and Foxp3^-^CD25^+^CD8^+^ aTeff cells in the cultures treated with MPA constituted about 50% of corresponding control values, and the mean absolute count of these cells constituted only about 20% and 45% of corresponding control values, respectively.

MPA-induced increase in Foxp3 expression in CD25^+^CD4^+^ and CD25^+^CD8^+^ T cells only to a small extent was responsible for the decline in the percentage of Foxp3^-^CD25^+^CD4^+^ and Foxp3^-^CD25^+^CD8^+^ aTeff cells. This is proven by the fact that MPA: (a) increased the percentage of Foxp3^+^CD25^+^CD4^+^ T cells on average by 5.54% (MPA 10^-4^ M) and 6.01% (MPA 10^-5^ M), but decreased the percentage of Foxp3^-^CD25^+^CD4^+^ T cells on average by 14.84% (MPA 10^-4^ M) and 13.48% (MPA 10^-5^ M); (b) increased the percentage of Foxp3^+^CD25^+^CD8^+^ T cells on average by 0.99% (MPA 10^-4^ M) and 0.87% (MPA 10^-5^ M), but decreased the percentage of Foxp3^-^CD25^+^CD8^+^ T cells on average by 21.39% (MPA 10^-4^ M) and 22.40% (MPA 10^-5^ M). Thus, the confrontation of these data clearly proves that MPA reduced the activation-induced CD25 expression on CD4^+^ and CD8^+^ T cells. Summarizing, the results indicate that MPA under activation conditions up-regulates Foxp3 expression in CD4^+^ and CD8^+^ T cells and down-regulates CD25 expression on these cells.

A natural consequence of the MPA-induced effects described above was the increase of both CD4^+^ Treg/CD4^+^ aTeff (**Figure 6I**) and CD8^+^ Treg/CD8^+^ aTeff cell ratios (**Figure 6J**). The mean value of CD4^+^ Treg/CD4^+^ aTeff and CD8^+^ Treg/CD8^+^ aTeff cell ratios in the cultures treated with MPA increased by about 4.5- and 10-times, respectively, compared to corresponding control values. The statistical analysis did not find any significant differences between both concentrations of MPA in terms of the parameters discussed above.

### 3.5 Administration of MMF induces an increase in the percentage of Foxp3^+^CD25^+^CD4^+^ and Foxp3^+^CD25^+^CD8^+^ T cells in the HNLNs and MLNs

**Figure 7:**
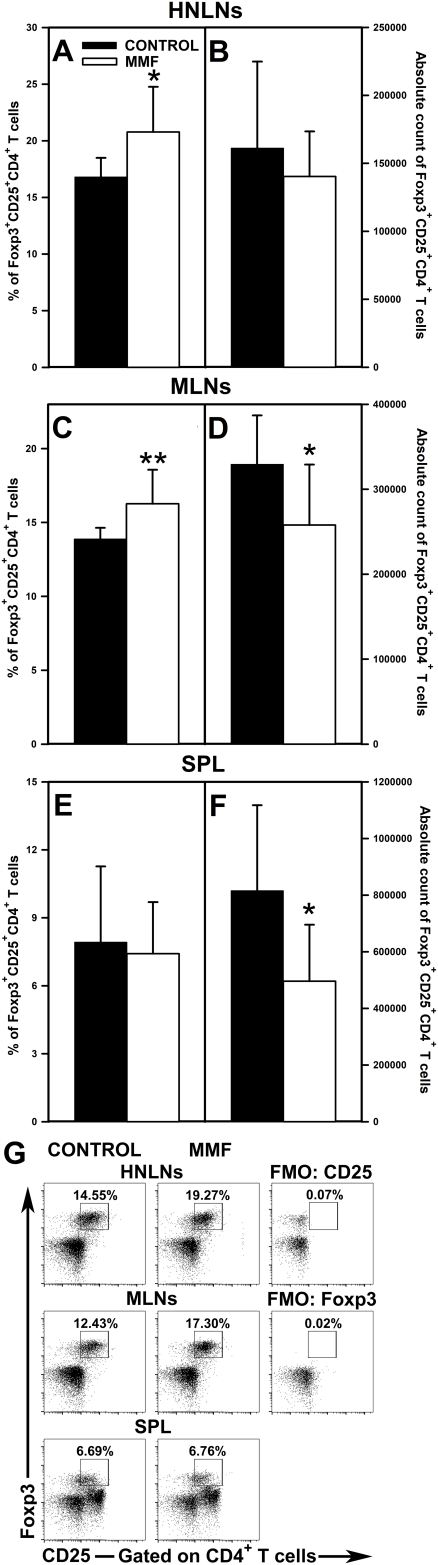
Effect of mycophenolate mofetil (MMF) on the percentage and absolute count of Foxp3^+^CD25^+^CD4^+^ regulatory T cells in the head and neck lymph nodes (HNLNs), mesenteric lymph nodes (MLNs) and spleen (SPL) in mice. The parameters were determined in the HNLNs, MLN and SPL of non-treated (CONTROL) and MMF-treated (MMF) mice. The relative count is expressed as a percentage of Foxp3^+^CD25^+^ cells within the CD4^+^ T cell subset **(A, C, E)**. The absolute count represents the number of Foxp3^+^CD25^+^CD4^+^ T cells in the whole HNLNs **(B)**, MLNs **(D)** and SPL **(F)** collected from individual mice. Results are presented as the mean (± S.D.) of two independent experiments with 5 mice per group (n = 10 per group, *P < 0.05, **P < 0.01). Examples of dot plot cytograms showing the distribution of Foxp3^+^CD25^+^ cells within the CD4^+^ T cell subsets **(G)**. Fluorescence minus one (FMO) controls were applied to confirm the gating strategy used to identify the Foxp3^+^CD25^+^ cell subset.

**Figure 8:**
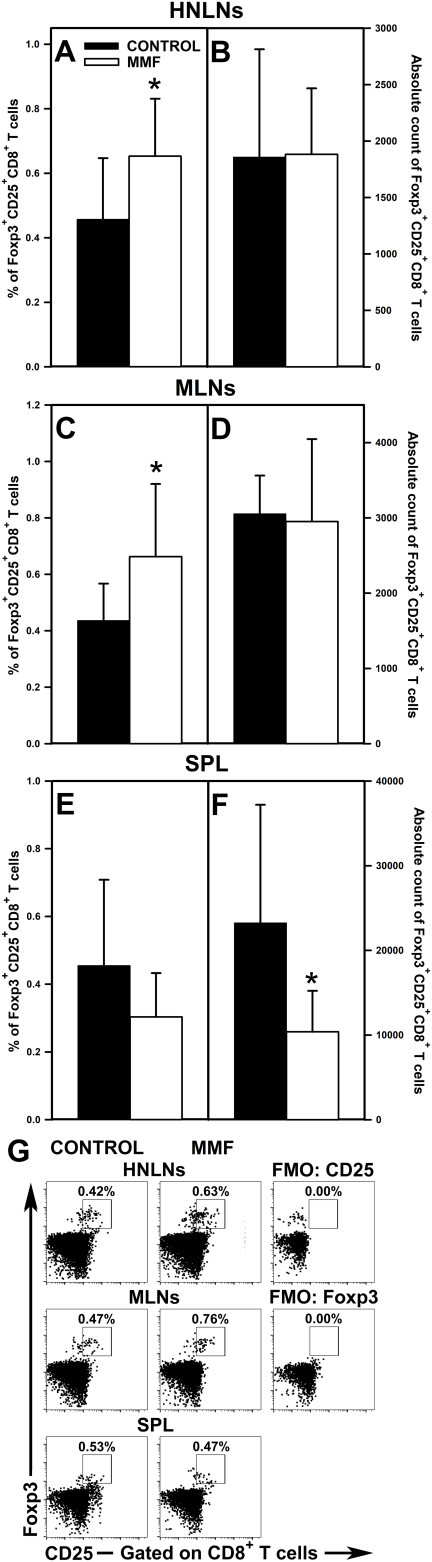
Effect of mycophenolate mofetil (MMF) on the percentage and absolute count of Foxp3^+^CD25^+^CD8^+^ regulatory T cells in the head and neck lymph nodes (HNLNs), mesenteric lymph nodes (MLNs) and spleen (SPL) in mice. The parameters were determined in the HNLNs, MLN and SPL of non-treated (CONTROL) and MMF-treated (MMF) mice. The relative count is expressed as a percentage of Foxp3^+^CD25^+^ cells within the CD8^+^ T cell subset **(A, C, E)**. The absolute count represents the number of Foxp3^+^CD25^+^CD8^+^ T cells in the whole HNLNs **(B)**, MLNs **(D)** and SPL **(F)** collected from individual mice. Results are presented as the mean (± S.D.) of two independent experiments with 5 mice per group (n = 10 per group, *P < 0.05). Examples of dot plot cytograms showing the distribution of Foxp3^+^CD25^+^ cells within the CD8^+^ T cell subsets **(G)**. Fluorescence minus one (FMO) controls were applied to confirm the gating strategy used to identify the Foxp3^+^CD25^+^ cell subset.

The research revealed that the administration of MMF led to a significant increase in the percentage of Foxp3^+^CD25^+^CD4^+^ T cells in the HNLNs (**Figure 7A** and upper panel of **Figure 7G**) and MLNs (**Figure 7C** and middle panel of **Figure 7G**), and did not affect the value of this parameter in the spleen (**Figure 7E** and lower panel of **Figure 7G**). However, the magnitude of these effects was relatively modest as the mean percentage of Foxp3^+^CD25^+^CD4^+^ cells in the HNLNs and MLNs was increased only by about 23% and 17%, respectively. These effects were not reflected by the corresponding increases in the absolute values. What is more, the treatment with MMF induced a decrease in the absolute count of Foxp3^+^CD25^+^CD4^+^ cells in the MLNs (**Figure 7D**) and spleen (**Figure 7F**), but did not affect the value of this parameter in the HNLNs (**Figure 7B**). Very similar results were obtained for Foxp3^+^CD25^+^CD8^+^ T cells. Administration of MMF increased the percentage of Foxp3^+^CD25^+^CD8^+^ T cells in the HNLNs (**Figure 8A** and upper panel of **Figure 8G**) and MLNs (**Figure 8C** and middle panel of **Figure 8G**), but did not affect the absolute count of these cells (**Figures 8B, D**). The mean percentage of Foxp3^+^CD25^+^CD8^+^ T cells in the HNLNs and MLNs was increased by about 43% and 52%, respectively. In turn, in the spleen of mice treated with MMF the percentage of these cells was not affected (**Figure 8E** and lower panel of **Figure 8G**) and their absolute count was considerably reduced (**Figure 8F**). A tabular summary of the results is shown in **Supplementary Table S1**.

## 4 Discussion

### 4.1 Evaluation of the effect of MMF/MPA on T and B cells in the context of undesirable effects

The present research has revealed that exposure of unstimulated cell cultures to MPA induced the loss of CD4^+^ and CD8^+^ T cells, and B cells. Similarly, the administration of MMF to healthy mice caused depletion of these cells in both evaluated lymph nodes and the spleen. When one compares the mean depletions of CD4^+^ and CD8^+^ T cells (including their IFN-γ-producing cell subsets, i.e. Th1 and Tc1 cells, respectively), and B cells induced by exposure to MPA with those caused by MMF in lymph nodes and the spleen, it can be stated that the scale of this effect was comparable between *in vitro* and *in vivo* conditions. These results clearly demonstrate that MMF may have a depletive effect not only on pathological T and B cells, but also on normal ones. Therefore, it can be concluded that MMF may exert destructive effects on lymphocytes, which play a leading role in the development and maintenance of humoral and cellular immunity.

In light of the available literature, this study appears to have been the first one ever focused on the effect of MMF and MPA on absolute counts of T and B cells in lymphoid tissues and cultures, respectively, hence no adequate data for comparison could be retrieved. However, a study in which the above parameters were analyzed in blood has been reported. In it, it was demonstrated that the treatment with MMF of patients with systemic lupus erythematosus reduced the absolute count of CD4^+^ T cells, CD8^+^ T cells and B cells in peripheral blood by about 49%, 35% and 48%, respectively (20). These results are relatively close to the ones presented in this paper.

The research demonstrated that MPA reduced the absolute count of IFN-γ-producing CD4^+^ and CD8^+^ T cells, i.e. Th1 and Tc1 cells, respectively. However, very importantly, the drug did not affect the frequency of CD4^+^ and CD8^+^ T cells capable of IFN-γ production. As it was previously explained (25), such results should be interpreted as the lack of direct impact of the drug on the ability of these cells to produce IFN-γ. Thus, these results indicate that MPA did not cause the loss of capacity of T cells to produce IFN-γ. In view of this, and taking into consideration the proapoptotic and strong antiproliferative effect of MPA toward CD4^+^ and CD8^+^ T cells demonstrated in this research, it can be concluded that the drug reduces the number of IFN-γ-producing Th1 and Tc1 cells most likely only in an indirect fashion, i.e. through the induction of apoptosis and inhibition of proliferation of these cells. In the nature of things, a reduction in the number of IFN-γ-producing Th1 and Tc1 cells should translate into decreased IFN-γ production. This assumption is consistent with the findings by He et al. (16), who found a decreased level of IFN-γ in the supernatant from cultures of T cells exposed to MPA.

MPA- or MMF-induced losses of B cells and particular subsets of T cells were statistically significant, although in any study of this kind it is proper to ask a question about the clinical relevance of the demonstrated effects. For peripheral blood, there are approved ranges of reference values for each type of lymphocytes, which can be used for reference when identifying the clinical significance of the loss of these cells under the impact of a tested factor. However, for an obvious reason, there are no such reference values applicable to lymph nodes and other lymphoid tissues. It is therefore impossible to determine whether the decreased counts of CD4^+^ and CD8^+^ T cells, and B cells in lymph nodes and the spleen of MMF-treated mice were within or below the normal range of corresponding reference values. Having averaged the losses of CD4^+^ and CD8^+^ T cells, and B cells counted for all the analyzed tissues, it was possible to conclude that treatment with MMF caused reduction of these cells by an average of 32.36%, 31.58% and 38.52%, respectively; exposure of cells on MPA caused losses of IFN-γ-producing CD4^+^ and CD8^+^ T cells on a comparable scale. Considering the key importance of T and B cells in the development and maintenance of effective immune protection, in the authors’ opinion, the drug-induced depletion of T and B cells in lymphoid tissues by more than 30% and nearly 40%, respectively, should be considered as having clinical relevance for the efficient humoral and cellular immune responses to infectious pathogens, and also vaccination efficacy. Therefore, the findings of this investigation can improve the benefit-risk assessment of using MMF in patients, especially those with bacterial and viral infections and undergoing vaccination.

### 4.2 Effects of MMF/MPA on T and B cells, which can be considered in the aspect of both the undesirable effects and immune mechanisms that may be responsible for clinical efficacy of the drug

It is widely accepted that the antiproliferative effect on T and B cells, exerted via IMPDH inhibition, constitutes the mechanism responsible for the immunosuppressive properties of MMF. However, the present study contributed new insights into this mechanism. Namely, the administration of MPA to the medium prior to culture almost completely abrogated the proliferation of the studied cells. Conversely, when MPA was added to the medium for the last 12 h, i.e. after lymphocytes had been activated and proliferated extensively, the agent exerted only a weak antiproliferative effect on T and B cells. These results indicate that MMF effectively prevents the proliferation of T and B cells, but may be less effective against already proliferating (i.e. activated) ones. The clinical implication of the finding is that to ensure the optimal clinical effectiveness of MMF, treatment should be started prior to the expected activation of T and/or B cells.

The present research indicates that additional (i.e. other than IMPDH inhibition) mechanisms may be involved in the development of the immunosuppressive properties of MMF. One of them is closely related to the depletive effect of MMF on T and B cells. A question arises about the mechanism behind the MMF/MPA-induced depletion of T and B cells revealed in our study. As the depletion of studied cells was demonstrated in non-proliferating cultures, the reason for it must have been increased cell death. In fact, the study found elevated apoptosis of CD4^+^ and CD8^+^ T cells, and B cells in the cultures exposed to the higher concentration of MPA. The effect might be seen as not spectacular, only slightly expressed, yet it was detectable. This is the first report showing that MMF may exert proapoptotic action on normal (i.e. non-activated) CD4^+^ and CD8^+^ T cells, and B cells. The depletive effect of MMF demonstrated under *in vivo* conditions was most likely not a consequence of the redistribution of T and B cells from the HNLNs, MLNs and spleen to other immune compartments, as their loss was also demonstrated under *in vitro* conditions. It should be assumed that proapoptotic and/or antiproliferative actions of MMF were responsible for its depletive effect on T and B cells under *in vivo* conditions.

If MMF exerts proapoptotic action on normal T and B cells, a possibility should be considered that the induction of apoptosis of Teff and B cells involved in the pathogenesis of autoimmune disorders may constitute an additional mechanism underlying the immunosuppressive properties of MMF. This assumption is supported by results of Izeradjene and Revillard (11), who demonstrated that MPA exerted a proapoptotic effect toward activated CD4^+^ and CD8^+^ T cells.

Although CD25 is typically associated with the Treg cell phenotype, CD25 is extensively expressed by effector T cells upon activation. The activation of CD4^+^ and CD8^+^ T cells is associated with early synthesis of IL-2 and upregulation of CD25; CD25 is the α-chain (IL-2Rα) of the receptor complex for IL-2 that confers high-affinity binding to IL-2. It enables the effective binding of IL-2 to effector T cells, increasing their responsiveness to IL-2 (26). Taking into account a pivotal role of IL-2 in orchestrating immune responses, it can be stated that the expression of CD25 plays a key role in regulating the intensity of proliferation of T cells and their differentiation T cells into subsets of effector and memory T cells. The research showed that MPA down-regulated or prevented the activation-induced CD25 expression on CD4^+^ and CD8^+^ Teff cells. This is consistent with the results of Weigel et al. (12), who reported that the treatment with MMF decreased the percentage of CD25-expressing T cells in patients after heart transplantation. In contrast, other studies did not found the effect of MPA on CD25 expression on peripheral blood lymphocytes (27), or the effect of MMF treatment on CD25 expression on T cells in patients with systemic lupus erythematosus (28).

The down-regulation of the CD25 expression on Teff cells makes them unresponsive to IL-2, which may limit autocrine and paracrine IL-2 dependent functions, including T-helper activities, cellular cytotoxicity and, above all, T cell proliferation (26, 29). Therefore, MPA-mediated down-regulation or prevention of induction of CD25 expression on Teff cells should be considered as an immunosuppressive action. This action may have a Janus face. On the one hand, the down-regulation of CD25 expression on Teff cells makes them less responsive to IL-2, which in consequence may compromise the normal immune response to pathogens and vaccines. On the other hand, it may attenuate the pathological immune response in the course of autoimmune diseases. What is more, although it is widely accepted that the antiproliferative effect of MMF involves the direct inhibition of IMPDH, considering the role of CD25 in T cell proliferation, the present results indicate that the drug may also indirectly, i.e. *via* down-regulation of CD25 on T cells, inhibit the proliferation of these cells. This assertion is supported by the results achieved in our study as it was found that the agent exerted only a weak antiproliferative effect on activated T and B cells. To conclude, the obtained results strongly suggest that the down-regulation or prevention of activation-induced CD25 expression on Teff cells (and maybe B cells) may be considered as additional mechanisms involved in producing the immunosuppressive properties of MMF.

The research demonstrated that MMF and MPA increased the percentage of Foxp3^+^CD25^+^CD4^+^ and Foxp3^+^CD25^+^CD8^+^ T cell subsets. Under the *in vitro* activation conditions, this effect was specially strongly expressed as the percentage of these cell subsets were increased 2- and 4-times, respectively. This effect was also found in lymph nodes of mice treated with MMF, although its magnitude was relatively modest (especially with respect to Foxp3^+^CD25^+^CD4^+^ T cells) compared to that observed under activation conditions. The MPA-induced increase in the percentage of Foxp3^+^CD25^+^CD8^+^ T cells was accompanied by an increase in the absolute number of these cells. However, in the remaining cases, MMF and MPA either reduced or left unaltered the absolute counts of these cells, which was most probably linked to the depletive effect of the drug on T cells. Notwithstanding this, the results clearly indicate that treatment with MMF may shift the Treg cell/aTeff cell balance toward an increased proportion of Treg cells. There are contradictory results from studies related to the impact of MMF or MPA on Foxp3 expression. Some research results suggest that MPA may increase Foxp3 expression in T cells (15, 16). In turn, other *in vitro* studies did not detect any effect of MPA on Foxp3 expression (14), or else the agent decreased the percentage of Foxp3^+^CD25^+^CD4^+^ T cells (19, 20). To conclude, the results of the present study strongly suggest that MMF may induce Foxp3 expression in Foxp3-negative CD4^+^ and CD8^+^ T cells, and thereby induce/promote conversion of conventional CD4^+^ and CD8^+^ T cells into iTreg. Thus, another mechanism through which MMF exerts its immunosuppressive effect may consist of inducing the generation of Foxp3-expressing CD4^+^ and CD8^+^ iTreg cells. And what’s more, in the light of these results, MMF may serve as a potential tool for the pharmacological induction of Foxp3-expressing CD4^+^ and CD8^+^ Treg cells. The findings can have two opposite clinical implications. On the one hand, the induction of Treg cells is currently perceived as a promising therapeutic strategy for the treatment of disorders associated with Treg cell deficiency or dysfunction. On the other hand, the generation of these cells may be considered as an undesired side effect, at least in oncologic patients, as both Foxp3^+^CD25^+^CD4^+^ (30) and Foxp3^+^CD25^+^CD8^+^ T cells (31, 32) inhibit the anti-cancer immunity, i.e. they contribute to immune response evasion against cancer and consequently progression of the disease.

The present study updated and extended the existing knowledge and understanding of the effects of MMF on T and B cells in terms of both safety and immune mechanisms underlying its therapeutic actions. In summary, the study found that the treatment of mice with MMF induced depletion of normal CD4^+^ and CD8^+^ T cells and B cells in lymph nodes and the spleen, and the magnitude of these effects should be considered as clinically relevant. These new data should be taken into account when assessing the benefit-risk ratio of using MMF in patients with bacterial and viral infections and undergoing vaccination. Moreover, they lead us to believe that the main cause of increased susceptibility to and severity of microbial infections in patients treated with MMF is the depletive/destructive effect of the drug on T and B cells in lymphoid tissue. The obtained results indicate that MMF effectively prevents the proliferation of T and B cells, but may be less effective against already proliferating cells. The findings of the study strongly suggest the existence of additional immune mechanisms that may be responsible for the clinical efficacy of the drug. These mechanisms may include the following: (a) proapoptotic action on CD4^+^ and CD8^+^ Teff cells, and B cells; (b) the down-regulation or prevention of activation-induced expression of CD25 on CD4^+^ and CD8^+^ Teff cells; both mechanisms shall be related to cells involved in the pathogenesis of autoimmune disorders; (c) the generation of CD4^+^ and CD8^+^ iTreg cells - via induction of Foxp3 expression in Foxp3-negative CD4^+^ and CD8^+^ T cells - resulting in the shift of the Treg cell/aTeff cell balance toward an increased proportion of Treg cells. However, all these effects may also be perceived as an undesired side effects because a proapoptotic action on normal Teff and B cells and the down-regulation of CD25 expression on these cells may compromise the immune response to pathogens and vaccines. In turn, Foxp3-expressing CD4^+^ and CD8^+^ Treg cells are known to inhibit the anti-cancer immunity.

## 5 Limitation of the study

In drug studies, the generalizability from animals to humans is often limited because of biological differences that exist between different species. Therefore, the primary limitation of this study was that the test subjects were mice and therefore these results may not translate perfectly to humans and other higher mammalian species. The second limitation of this study is that the sample size is relatively small (n = 10 or 15), which might limit the robustness and generalizability of the findings. The third limitation of the research is the short length of the study period. In order to obtain more reliable results, the study should be replicated with larger number of mice treated with MMF for longer period of time.

## Data Availability

The raw data supporting the conclusions of this article will be made available by the authors, without undue reservation.
