# Supplementary material for: Mycophenolate mofetil: an update on its mechanism of action and effect on lymphoid tissue
Source: Front Immunol. 2025 Jan 7;15:1463429. doi: 10.3389/fimmu.2024.1463429 (PMC11753233; doi:10.3389/fimmu.2024.1463429)
Supplement: Supplementary file 1 [file DataSheet1.docx]

Supplementary Materials

Mycophenolate mofetil: an update on its mechanism of action and effect on lymphoid tissue

Anna Krawczyk, Bernard Kravčenia, Tomasz Maślanka*





**Supplementary Figure S1.** Gating strategy for FACS analysis and calculation of the absolute cell counts of lymphocyte subsets. (A) Identification of T cell subsets and B cells. Lymphocytes were identified based on forward and side scatter (FSC/SSC) properties, and then gated for expression of CD4, CD8 or CD19 surface receptors. CD4^+^ and CD8^+^ T cells were analyzed for expression/co-expression of CD25 and Foxp3. On this basis, the CD4^+^ and CD8^+^ T cell populations were subdivided into regulatory [Foxp3^+^CD25^+^CD4^+^ (CD4^+^ Treg) and Foxp3^+^CD25^+^CD8^+^ (CD8^+^ Treg) cells] and activated effector [Foxp3^-^CD25^+^CD4^+^ (CD4^+^ aTeff) and Foxp3^-^CD25^+^CD4^+^ (CD8^+^ aTeff) cells] subsets. Absolute cell counts of lymphocyte subsets [i.e. number of cells from particular subpopulations per organ (in vivo studies) or per well (in vitro studies)] were calculated using the dual platform method, as shown above. (B) Identification of proliferating and early apoptotic T and B cells. CD4^+^ and CD8^+^ T cells as well as B (i.e. CD19^+^) cells were analyzed for apoptosis by using Annexin V and 7-aminoactinomycin D (7-AAD) staining. Annexin V^-^7-AAD^-^ cells were considered viable Annexin V^+^7-AAD^-^ cells were deemed early-apoptotic and double positive cells (Annexin V^+^7-AAD^+^) were considered to be a mixture of late apoptotic and primary necrotic cells. Moreover, CD4^+^ and CD8^+^ T cells, and B cells were assessed for proliferation by measuring bromo-2′-deoxyuridine (BrdU) incorporation BrdU-positive cells were considered as proliferating cells.

**Supplementary Table S1.** Tabular summary of the results provided in the paper

|  | **CONTROL (CON)** | | **MPA 10-4** | | **MPA 10-4** | | ***P*-value** | | |
| --- | --- | --- | --- | --- | --- | --- | --- | --- | --- |
|  | **The mean** | **SD** | **The mean** | **SD** | **The mean** | **SD** | **CON vs. MPA 10^-4^** | **CON vs. MPA 10^-5^** | **MPA 10-4 vs. MPA 10^-5^** |
|  | **Effect of mycophenolic acid (MPA) on the absolute count of CD4^+^ and CD8^+^ T cells, and CD19^+^ cells under in vitro conditions** | | | | | | | | |
|  | **Absolute count of CD4^+^ T cells** | | | | | | | | |
| **48 h** | 1475407 | 297460 | 959260 | 189324 | 1079833 | 242862 | ≤ 0.001 | ≤ 0.001 | NS |
| **96 h** | 1188000 | 266407 | 579360 | 117750 | 630533 | 160290 | ≤ 0.001 | ≤ 0.001 | NS |
|  | **Absolute count of CD8^+^ T cells** | | | | | | | | |
| **48 h** | 375720 | 83137 | 243647 | 44222 | 286347 | 62905 | ≤ 0.001 | 0.002 | NS |
| **96 h** | 229847 | 63218 | 111413 | 25920 | 125053 | 38634 | ≤ 0.001 | ≤ 0.001 | NS |
|  | **Absolute count of CD19^+^ cells** | | | | | | | | |
| **48 h** | 376007 | 86998 | 250020 | 70464 | 253053 | 73626 | ≤ 0.001 | ≤ 0.001 | NS |
| **96 h** | 73853 | 30835 | 33299 | 11286 | 35155 | 17692 | ≤ 0.001 | ≤ 0.001 | NS |
|  | **Effect of MPA on the relative and absolute counts of IFN-γ-producing CD4^+^ and CD8^+^ T cells** | | | | | | | | |
|  | **% of IFN-γ-producing CD4^+^ T cells (Th1 cells)** | | | | | | | | |
|  | 7.78 | 2.56 | 7.78 | 2.41 | 7.34 | 2.40 | NS | NS | NS |
|  | **% of IFN-γ-producing CD8^+^ T cells (Tc1 cells)** | | | | | | | | |
|  | 4.42 | 0.99 | 4.08 | 1.44 | 4.25 | 1.01 | NS | NS | NS |
|  | **Absolute count of IFN-γ-producing CD4^+^ T cells (Th1 cells)** | | | | | | | | |
|  | 26963 | 5898 | 18528 | 5167 | 19410 | 6427 | ≤ 0.001 | < 0.01 | NS |
|  | **Absolute count of IFN-γ-producing CD8^+^ T cells (Tc1 cells)** | | | | | | | | |
|  | 24080 | 7274 | 13673 | 6393 | 16664 | 6175 | ≤ 0.001 | < 0.05 | NS |
|  | **Effect of MPA on proliferation of CD4+ and CD8+ T cells, and CD19+ cells** | | | | | | | | |
|  | **% of BrdU-incorporating CD4^+^ T cells** | | | | | | | | |
| **78 h** | 33.35 | 6.80 | 3.34 | 2.39 | 3.66 | 1.96 | ≤ 0.001 | ≤ 0.001 | NS |
| **Last 12 h** | 38.05 | 5.46 | 31.01 | 5.03 | 32.40 | 5.31 | < 0.05 | NS (0.071) | NS |
|  | **% of BrdU-incorporating CD8^+^ T cells** | | | | | | | | |
| **78 h** | 46.07 | 6.39 | 5.73 | 5.41 | 8.20 | 6.67 | ≤ 0.001 | ≤ 0.001 | NS |
| **Last 12 h** | 48.90 | 8.31 | 38.12 | 9.71 | 40.48 | 8.77 | < 0.05 | NS | NS |
|  | **% of BrdU-incorporating CD19^+^ cells** | | | | | | | | |
| **78 h** | 52.53 | 10.26 | 6.80 | 7.19 | 9.75 | 9.01 | ≤ 0.001 | ≤ 0.001 | NS |
| **Last 12 h** | 39.73 | 5.95 | 33.13 | 3.43 | 33.38 | 3.59 | < 0.01 | < 0.05 | NS |

| . | **CONTROL (CON)** | | **MPA 10-4** | | **MPA 10-4** | | ***P*-value** | | |
| --- | --- | --- | --- | --- | --- | --- | --- | --- | --- |
|  | **The mean** | **SD** | **The mean** | **SD** | **The mean** | **SD** | **CON vs. MPA 10^-4^** | **CON vs. MPA 10^-5^** | **MPA 10-4 vs. MPA 10^-5^** |
|  | **Effect of mycophenolic acid (MPA) on apoptosis of CD4^+^ and CD8^+^ T cells, and CD19^+^ cells** | | | | | | | | |
|  | **% of early apoptotic CD4^+^ T cells** | | | | | | | | |
| **24 h** | 1.03 | 0.26 | 1.45 | 0.37 | 1.31 | 0.32 | < 0.05 | NS | NS |
| **48 h** | 0.81 | 0.13 | 1.09 | 0.26 | 0.89 | 0.13 | < 0.01 | NS | NS |
|  | **% of early apoptotic CD8^+^ T cells** | | | | | | | | |
| **24 h** | 1.26 | 0.31 | 1.92 | 0.63 | 1.46 | 0.34 | < 0.01 | NS | NS |
| **48 h** | 0.82 | 0.12 | 0.85 | 0.08 | 0.87 | 0.18 | NS | NS | NS |
|  | **% of early apoptotic CD19^+^ T cells** | | | | | | | | |
| **24 h** | 3.74 | 1.02 | 5.93 | 2.12 | 6.17 | 2.13 | < 0.05 | < 0.05 | NS |
| **48 h** | 6.47 | 2.73 | 6.24 | 0.96 | 7.44 | 2.91 | NS | NS | NS |
|  | **Effect of MPA on the relative and absolute counts of Foxp3^+^CD25^+^CD4^+^, Foxp3^+^CD25^+^CD8^+^ T regulatory (CD4^+^ and CD8^+^ Treg, respectively) cells and Foxp3^-^CD25^+^CD4^+^ and Foxp3^-^CD25^+^CD8^+^ activated effector T (CD4^+^ and CD8^+^ aTeff, respectively)** | | | | | | | | |
|  | **% of Foxp3^+^CD25^+^CD4^+^ T cells (CD4^+^ Treg cells)** | | | | | | | | |
|  | 4.77 | 1.14 | 10.32 | 3.15 | 10.78 | 3.17 | < 0.001 | < 0.001 | NS |
|  | **% of Foxp3^+^CD25^+^CD8^+^ T cells (CD8^+^ Treg cells)** | | | | | | | | |
|  | 0.28 | 0.11 | 1.27 | 0.40 | 1.14 | 0.41 | < 0.001 | < 0.001 | NS |
|  | **Absolute count of Foxp3^+^CD25^+^CD4^+^ T cells (CD4^+^ Treg cells)** | | | | | | | | |
|  | 37979 | 9791 | 31074 | 11205 | 33827 | 10225 | NS | NS | NS |
|  | **Absolute count of Foxp3^+^CD25^+^CD8^+^ T cells (CD8^+^ Treg cells)** | | | | | | | | |
|  | 846 | 470 | 3086 | 963 | 2891 | 720 | < 0.001 | < 0.001 | NS |
|  | **% of Foxp3^-^CD25^+^CD4^+^ cells (CD4^+^ aTeff cells)** | | | | | | | | |
|  | 29.62 | 6.56 | 14.76 | 5.32 | 16.14 | 5.70 | < 0.001 | < 0.001 | NS |
|  | **% of Foxp3^-^CD25^+^CD8^+^ T cells (CD8^+^ aTeff cells)** | | | | | | | | |
|  | 40.65 | 7.65 | 19.25 | 4.65 | 18.24 | 5.13 | < 0.01 | < 0.001 | NS |
|  | **Absolute count of Foxp3^-^CD25^+^CD4^+^ T cells (CD4^+^ aTeff cells)** | | | | | | | | |
|  | 234919 | 49990 | 43714 | 16029 | 49498 | 13353 | < 0.001 | < 0.001 | NS |
|  | **Absolute count of Foxp3^-^CD25^+^CD8^+^ T cells (CD8^+^ aTeff cells)** | | | | | | | | |
|  | 131997 | 59387 | 51667 | 23678 | 49692 | 18335 | < 0.001 | < 0.001 | NS |
|  | **CD4^+^ Treg/CD4^+^ aTeff cell ratio** | | | | | | | | |
|  | 0.17 | 0.05 | 0.77 | 0.33 | 0.75 | 0.35 | < 0.001 | < 0.001 | NS |
|  | **CD8^+^ Treg/CD8^+^ aTeff cell ratio** | | | | | | | | |
|  | 0.0071 | 0.0030 | 0.0700 | 0.0329 | 0.0793 | 0.0715 | < 0.001 | < 0.001 | NS |

|  | **CONTROL** | | **MMF** | | ***P*-value** |
| --- | --- | --- | --- | --- | --- |
|  | **The mean** | **SD** | **The mean** | **SD** |  |
|  | **Effect of mycophenolate mofetil (MMF) on the absolute count of CD4^+^ and CD8^+^ T cells, and CD19^+^ cells in the head and neck lymph nodes (HNLNs), mesenteric lymph nodes (MLNs) and spleen (SPL) in mice** | | | | |
|  | **Absolute count of CD4^+^ T cells** | | | | |
| **HNLNs** | 950175 | 314510 | 684200 | 144011 | < 0.05 |
| **MLNs** | 2374555 | 404937 | 1612400 | 488978 | < 0.01 |
| **SPL** | 10504000 | 1968100 | 6617200 | 1555965 | ≤ 0.001 |
|  | **Absolute count of CD8^+^ T cells** | | | | |
| **HNLNs** | 407925 | 144841 | 291200 | 69733 | < 0.05 |
| **MLNs** | 791235 | 225631 | 523170 | 170369 | < 0.01 |
| **SPL** | 5039920 | 902347 | 3413800 | 822755 | ≤ 0.001 |
|  | **Absolute count of CD19^+^ cells** | | | | |
| **HNLNs** | 1112950 | 343317 | 594650 | 107459 | ≤ 0.001 |
| **MLNs** | 1973150 | 414794 | 1378700 | 474159 | < 0.01 |
| **SPL** | 24639600 | 5540195 | 15066400 | 4310375 | ≤ 0.001 |
|  | **Effect of MMF on the percentage and absolute count of Foxp3^+^CD25^+^CD4^+^ and Foxp3^+^CD25^+^CD8^+^ regulatory T cells in tha HNLNs, MLNs and SPL in mice** | | | | |
|  | **% of Foxp3^+^CD25^+^CD4^+^ T cells (CD4^+^ Treg cells)** | | | | |
| **HNLNs** | 16.80 | 1.70 | 20.77 | 4.00 | < 0.05 |
| **MLNs** | 13.88 | 0.75 | 16.27 | 2.30 | < 0.01 |
| **SPL** | 7.92 | 3.35 | 7.41 | 2.28 | NS |
|  | **% of Foxp3^+^CD25^+^CD8^+^ T cells (CD8^+^ Treg cells)** | | | | |
| **HNLNs** | 0.46 | 0.19 | 0.65 | 0.18 | < 0.05 |
| **MLNs** | 0.44 | 0.13 | 0.66 | 0.26 | < 0.05 |
| **SPL** | 0.46 | 0.25 | 0.30 | 0.13 | NS |
|  | **Absolute count of Foxp3^+^CD25^+^CD4^+^ T cells (CD4^+^ Treg cells)** | | | | |
| **HNLNs** | 161180 | 63671 | 140348 | 33118 | NS |
| **MLNs** | 329314 | 57537 | 257848 | 71235 | < 0.05 |
| **SPL** | 815560 | 301850 | 495880 | 199493 | < 0.05 |
|  | **Absolute count of Foxp3^+^CD25^+^CD8^+^ T cells (CD8^+^ Treg cells)** | | | | |
| **HNLNs** | 1858 | 954 | 1882 | 585 | NS |
| **MLNs** | 3055 | 507 | 2951 | 1096 | NS |
| **SPL** | 23248 | 13940 | 10383 | 4834 | < 0.05 |
